# Complete Genome Sequence of *Streptomyces* Bacteriophage Abt2graduatex2

**DOI:** 10.1128/genomeA.01480-17

**Published:** 2018-01-18

**Authors:** Ivan Erill, Steven M. Caruso

**Affiliations:** a Department of Biological Sciences, University of Maryland, Baltimore County, Baltimore, Maryland, USA

## Abstract

The *Streptomyces* bacteriophage Abt2graduatex2 is a double-stranded DNA (dsDNA) *Siphoviridae* isolated from soil collected in Baltimore, MD, and harvested using *Streptomyces griseus* subsp. *griseus*. Abt2graduatex2, a cluster BG phage, encodes an HicA-like toxin.

## GENOME ANNOUNCEMENT

Presented here is the complete genome sequence of the *Streptomyces* phage Abt2graduatex2, a double-stranded DNA (dsDNA) *Siphoviridae* isolated from soil collected in Baltimore, MD (39°15′28.1″N, 76°42′23.5″W) and investigated using *Streptomyces griseus* subsp. *griseus* (ATCC 10137) as a host by the UMBC Phage Hunters as part of the SEA-PHAGES program (1). *Streptomyces* species are filamentous *Actinomycetes* known for the production of antibiotics and other bioactive compounds (2).

Incubation on lawns of *S. griseus* grown on supplemented nutrient agar (3) overnight at 28°C produced clear plaques approximately 1 mm in diameter. Examination of *Streptomyces* phage Abt2graduatex2 by transmission electron microscopy (TEM) showed that the phage has a prolate head 82 nm in length and 53 nm in width and a flexible, noncontractile, 179-nm-long tail.

Abt2graduatex2 crude lysate was unable to infect or lyse the *Streptomyces* species *S. azureus* (NRRL B-2655), *S. filamentosus* (NRRL B-5411), or *S. venezuelae* (NRRL ISP-5230), even at an approximate MOI of 1,000, and was unable to produce infectious particles in *S. scabiei* RL-34 (ATCC 49173), though it was able to lyse this potato pathogen at that concentration (4).

Sequencing was performed by the Pittsburgh Bacteriophage Institute to 808-fold coverage by Illumina sequencing and assembled using Newbler and Consed (5). Abt2graduatex2 has a circularly permuted chromosome that was linearized at the 5′ end of the most distal of two genes tightly linked to the predicted terminase gene as described previously (6). The Abt2graduatex2 genome is 57,385 bp in length, with a G+C content of 69.2% and 90.83% coding density. Genome annotation was completed using DNA Master (http://cobamide2.bio.pitt.edu). The phage has 71 protein-coding genes, with a start codon breakdown of 71.8% AUG and 28.2% GUG. No tRNA genes have been identified. Abt2graduatex2 is a member of cluster BG (7), with which it shares an average (±SD) nucleotide identity of 82.8% ± 5.5% (8) or 85.3% ± 1.0% excluding the outlier cluster BG phage Xkcd426, with which it shares an average nucleotide identity (ANI) of 73% (GenBank accession number KU530220).

Abt2graduatex2 gp29 has been identified as a putative class II holin (9) by structural homology (10) and SOSUI analysis showing two transmembrane regions (11). Abt2graduatex2 gp29 is present in all current members of the BG cluster, where it forms a putative operon with gp28, a lysin, but it is absent in all other *Actinobacteria* phages sequenced to date. Abt2graduatex2 gp67 has been found to encode an HicA-like toxin, also present in all other cluster BG phages (12). To date, no corresponding protein antitoxin has been identified, suggesting that the space between gp67 and gp68 may encode an RNA antitoxin. Additional details on Abt2graduatex2, Xkcd426, and other phages isolated by SEA-PHAGES undergraduate students can be found in the Actinobacteriophage Database (13).

### Accession number(s).

The complete genome sequence of the *Streptomyces* phage Abt2graduatex2 is available in GenBank with the accession number MF975638.
